# Artificial intelligence in dentistry: Assessing the informational quality of YouTube videos

**DOI:** 10.1371/journal.pone.0316635

**Published:** 2025-01-02

**Authors:** Sachin Naik, Abdulaziz Abdullah Al-Kheraif, Sajith Vellappally

**Affiliations:** Dental Health Department, College of Applied Medical Sciences, King Saud University, Riyadh, Saudi Arabia; University of Sharjah College of Dental Medicine, UNITED ARAB EMIRATES

## Abstract

**Background and purpose:**

The most widely used social media platform for video content is YouTube^TM^. The present study evaluated the quality of information on YouTube^TM^ on artificial intelligence (AI) in dentistry.

**Methods:**

This cross-sectional study used YouTube^TM^ (https://www.youtube.com) for searching videos. The terms used for the search were "artificial intelligence in dentistry," "machine learning in dental care," and "deep learning in dentistry." The accuracy and reliability of the information source were assessed using the DISCERN score. The quality of the videos was evaluated using the modified Global Quality Score (mGQS) and the Journal of the American Medical Association (JAMA) score.

**Results:**

The analysis of 91 YouTube™ videos on AI in dentistry revealed insights into video characteristics, content, and quality. On average, videos were 22.45 minutes and received 1715.58 views and 23.79 likes. The topics were mainly centered on general dentistry (66%), with radiology (18%), orthodontics (9%), prosthodontics (4%), and implants (3%). DISCERN and mGQS scores were higher for videos uploaded by healthcare professionals and educational content videos(P<0.05). DISCERN exhibited a strong correlation (0.75) with the video source and with JAMA (0.77). The correlation of the video’s content and mGQS, was 0.66 indicated moderate correlation.

**Conclusion:**

YouTube™ has informative and moderately reliable videos on AI in dentistry. Dental students, dentists and patients can use these videos to learn and educate about artificial intelligence in dentistry. Professionals should upload more videos to enhance the reliability of the content.

## Introduction

YouTube™ has established itself as a dominant social media platform, with its user base growing steadily. The platform’s vast variety of content appeals to people of all ages and interests, fueling its ongoing expansion. The ease with which users can join YouTube™ has attracted millions of content producers worldwide [1, 2]. In November 2022, computer users viewed approximately eight billion videos on YouTube™, while phone device users visited the site over 71.66 billion times. YouTube^TM^ users are projected to increase by 346 million (+44.05%) from 2022 to 2028 [3]. Artificial Intelligence(AI) has significantly impacted industries like healthcare, enhancing diagnostic and therapeutic decisions in recent years. AI is empowering patients in dentistry with information and preliminary evaluations.

John McCarthy provided the initial definition of AI, previously referred to as applied computer engineering in 1956. Today, AI is often associated with the ’fourth industrial revolution,’ using digital technology to simulate human-like judgment, reasoning, and intelligent behaviour [4, 5].

Educational resources should grow at the same rapid pace as AI in dentistry. The adoption of AI in dentistry is accelerating quickly and has shown great promise in enhancing efficiency, accuracy, and patient outcomes [6, 7]. YouTube™ excels at presenting complex information in an engaging and easy-to-understand format. Videos can illustrate intricate procedures and concepts related to AI in dentistry, making them more accessible than traditional academic literature. A previous study found that YouTube™ videos can reach a vast audience, with some videos related to oral health, gathering over 30,000 views. This indicates a strong public interest in dental education [8]. This reach is beneficial for disseminating information about emerging technologies like AI, which may not yet be widely covered in formal educational settings. YouTube™ is a widely accessible educational platform, offering engaging, visual content that enhances learning and knowledge retention, particularly in healthcare and dental education [9–11].

However, the educational content on platforms like YouTube™ may not always reflect the latest research findings or clinical applications, potentially leaving viewers with outdated or incomplete knowledge [12]. Previous research found that 70% of individuals who sought health-related information reported that the Internet influenced their decision-making process [13]. A previous study projected that the global healthcare AI market, including dental care, will reach $45.2 billion by 2026 [14]. A narrative review emphasizes the superiority of AI-assisted methodologies over conventional techniques in various dental disciplines [15].

AI is not yet ubiquitous in the dental profession, but technological advancements have revolutionized dentistry. Convolutional neural networks (CNNs) and 2D/3D photography are used in dentistry to create 3D dental prostheses, yielding promising outcomes [16]. AI applications have been utilized in various areas of dentistry, such as oral and maxillofacial surgery [17, 18], caries and endodontics [19, 20], periodontics [21], temporomandibular joint disorders [22], orthodontics [23, 24], prosthodontics [24, 25], implants [26], etc.

Critical analysis of the information provided will improve the quality of AI in the dentistry-related content published on YouTube™. Without strict peer review, the information available in the YouTube™ videos runs the great risk of misinformation. This aspect is of greater concern in health-related professions, as it could lead to poor clinical outcomes for patients. A previous study highlighted that many YouTube™ videos on health-related topics are of low quality, commercially oriented, and lack the evidence-based support [27]. The integration of AI in dentistry necessitates high-quality YouTube content to educate professionals and patients, fostering accurate understanding of AI’s applications in diagnosis, treatment planning, and decision-making. This study will bridge research gaps by evaluating the accuracy, reliability, and educational value of YouTube™ videos related to the application of AI in dentistry. This study will contribute to the growing body of knowledge about digital platforms for sharing health-related information. Therefore, the aim of the present study was to evaluate the quality of information on YouTube™ regarding the use of artificial intelligence in dentistry.

## Material and methods

### Search strategy

**Fig 1:**
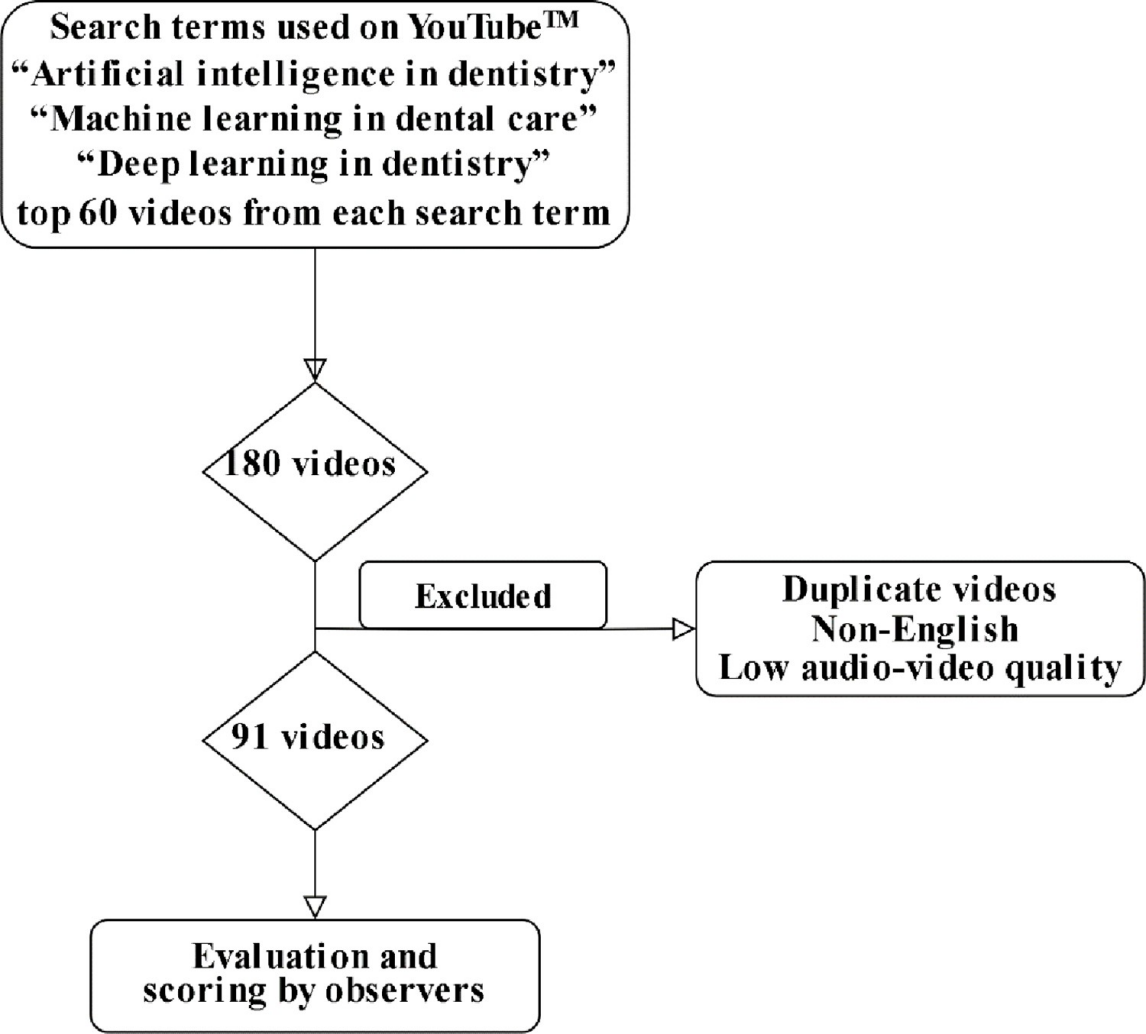
Flowchart illustrating the search results and screening process.

We used the online library source YouTube™ (https://www.youtube.com) to search for videos. On May 5, 2024, the search was conducted using the following search terms: (1) "artificial intelligence in dentistry," (2) "machine learning in dental care," and (3) "deep learning in dentistry". These terms were selected because they are highly specific to the research topic, encompass both the general concept and specific functionalities of AI in dentistry, and are also highly relevant to the search queries commonly used on YouTube^TM^ **(Fig 1).**

### Eligibility criteria

The study included English-language videos with good audio and video quality (720p HD). Videos in languages other than English and those with low audio or video quality were excluded. There were no restrictions on video duration or view count, and segmented videos were considered as one.

### Search execution

The search was conducted using a newly created YouTube™ account that had not been used before. This approach ensured that the YouTube™ algorithm presented content without being influenced by any previous user interactions [25]. The YouTube^TM^ search results were sorted using the ‘‘Relevance-Based Ranking” criteria [28], and no other filter was set.

### Video selection and storage

The top 60 videos for each search term were saved in the library. This decision was based on research indicating that 95% of individuals conducting internet searches view only the first three results pages [29]. Duplicate videos were removed from each category.

### Data collection

The title, posting date, number of likes, views, comments, and length of each video were recorded. The videos’ source URLs were saved to maintain the integrity of the research. This study utilized a dataset of publicly available YouTube^TM^ videos to evaluate content (S1 Data). All data collection and analysis methods strictly adhered to YouTube’s terms of service and community guidelines to ensure compliance with ethical and legal standards.

### Video categorization

The evaluated videos were categorized based on three main criteria: source, content type, and specialty.

### Source

- Healthcare Professionals: Content uploaded by health professionals.
- Independent Media: Videos from non-affiliated media sources or individuals.
- Commercial: Videos from commercial entities promoting products or services.

### Content type

- Information on AI: Videos discussing general concepts, benefits, limitations, or future developments of AI, specifically in dental contexts.
- Educational: Instructional videos that broadly cover AI topics without hands-on demonstrations or specific techniques.
- AI Teaching Techniques: Videos focusing on teaching methods, strategies, or tools for explaining AI applications.
- Software: Reviews or demonstrations of specific AI software, showcasing functionalities relevant to dental or healthcare applications.
- Advertisement: Promotional content for AI-related products or services, primarily focused on marketing.

### Specialty

- General
- Orthodontics
- Radiology
- Implants
- Prosthodontics

### Observer training and calibration process

Two observers, SN and SV, evaluated the videos for quality, reliability, and understandability. Both observers hold doctorate degrees and specialize in dental public health at King Saud University. A training and calibration process were conducted to ensure consistent evaluation of video content. This process included presentations and example videos to familiarize the observers with the DISCERN, JAMA, and mGQS assessment tools. Calibration exercises involved independent assessments followed by consensus-building discussions. Assessments were carried out before and after training on five videos. The intra-class correlation coefficient (ICC) for the evaluations of video content was calculated, yielding an ICC value of 0.90 (95% CI: 0.85–0.94), indicating excellent reliability among the observers [30]. This high level of agreement indicates that the scoring of video content was consistent across observers.

### Evaluation methods for video content

The “quality,” “accuracy,” and “reliability” of the YouTube^TM^ videos were assessed with specific tools to avoid overlap and ensure clarity. Detailed descriptions of the scoring criteria for each tool are provided in supportive information (S2 Data).

#### Quality

Quality is the overall standard of the information provided, assessed with DISCERN and mGQS. The DISCERN assessment tool was created for laypersons and is frequently used to evaluate the quality of information [31]. The DISCERN tool is a validated instrument designed to assess the quality and reliability of health information, focusing on aspects such as source credibility, transparency of information, clarity, and balance in presenting treatment options, including the discussion of benefits, risks, and alternatives. The tool comprises 16 questions divided into three sections. Each question was assessed using a 5-point Likert scale.

The modified Global Quality Index (mGQS) scale was used to assess video quality [32]. The mGQS, initially developed for assessing the educational value of online resources, has been adapted in various fields. However, its formal validation has not been documented. Despite this, mGQS was selected for this study due to its structured approach and applicability to digital video content. The tool rates videos based on comprehensiveness, clarity, and educational value, which align with the study’s objectives. This scale rating ranges from 1 to 5, with 1 representing poor quality, 2 generally poor quality, 3 moderate quality, 4 good quality, and 5 excellent quality.

#### Accuracy

Accuracy refers to factual correctness and alignment with current evidence, assessed by the Journal of American Medical Association (JAMA). Each video source was evaluated based on the JAMA benchmark standards [33]. Each accepted criteria were assigned one point, resulting in a total score ranging from 0 to 4 (1- Authorship, 2- Attribution, 3- Disclosure, 4- Currency), with a score of four indicating higher quality. This framework examines four critical components: authorship, ensuring the credibility of the author; attribution, confirming proper source citation; disclosure of conflicts of interest; and currency, assessing the timeliness of the information.

#### Reliability

Reliability is the trustworthiness of the information source, assessed by JAMA and DISCERN.

The research was exempt from approval by an institutional research ethics review board, as it only used publicly accessible data. No personal data was collected from the videos or comments during the study.

### Statistical analysis

The two observers used a Microsoft Office Excel spreadsheet to collect the data separately. Descriptive statistics were generated for each video, such as the number of views, length, comments, and likes. Frequencies and percentages were calculated for categorical data, while mean and range were computed for continuous variables. The ANOVA test was used to find an association between the groups. ICC was used for measuring reliability or consistency among raters. Correlation analysis using Pearson’s method was performed to test for associations with a significance level of 0.05. SPSS IBM version 25 was used for the data analysis.

## Results

The study analyzed 91 YouTube™ videos on AI use in dentistry. The average video length was 22.45 minutes (SD = 27.31), with a median of 7.25 minutes (1–89.11). Videos had an average of 1715.58 views (SD = 3460.61). The average number of comments was 1.22 (SD = 1.8). Videos received an average of 23.79 likes (SD = 76.02). The like ratio averaged 1.87% (SD = 2.62). Channels had an average of 6058.75 subscribers (SD = 13,632.47) (**Table 1**).

**Table 1:** Quantitative characteristics of YouTube ^TM^ videos on AI use in dentistry (n = 91).

Characteristics of videos	Mean (SD)	Median (Min-Max)
Length video(minutes)	22.45 (27.31)	7.25(1–89.11)
Views	1715.58(3460.61)	501(28–22575)
Number of comments	1.22(1.8)	0(0–9)
Number of likes	23.79 (76.02)	7(0–702)
Like ratio (likes/views) ×100	1.87(2.62)	1.39(0–19.23)
Number of subscribers	6058.75 (13632.469)	1340(8–8200)

SD-Standard Deviation

**Fig 2:**
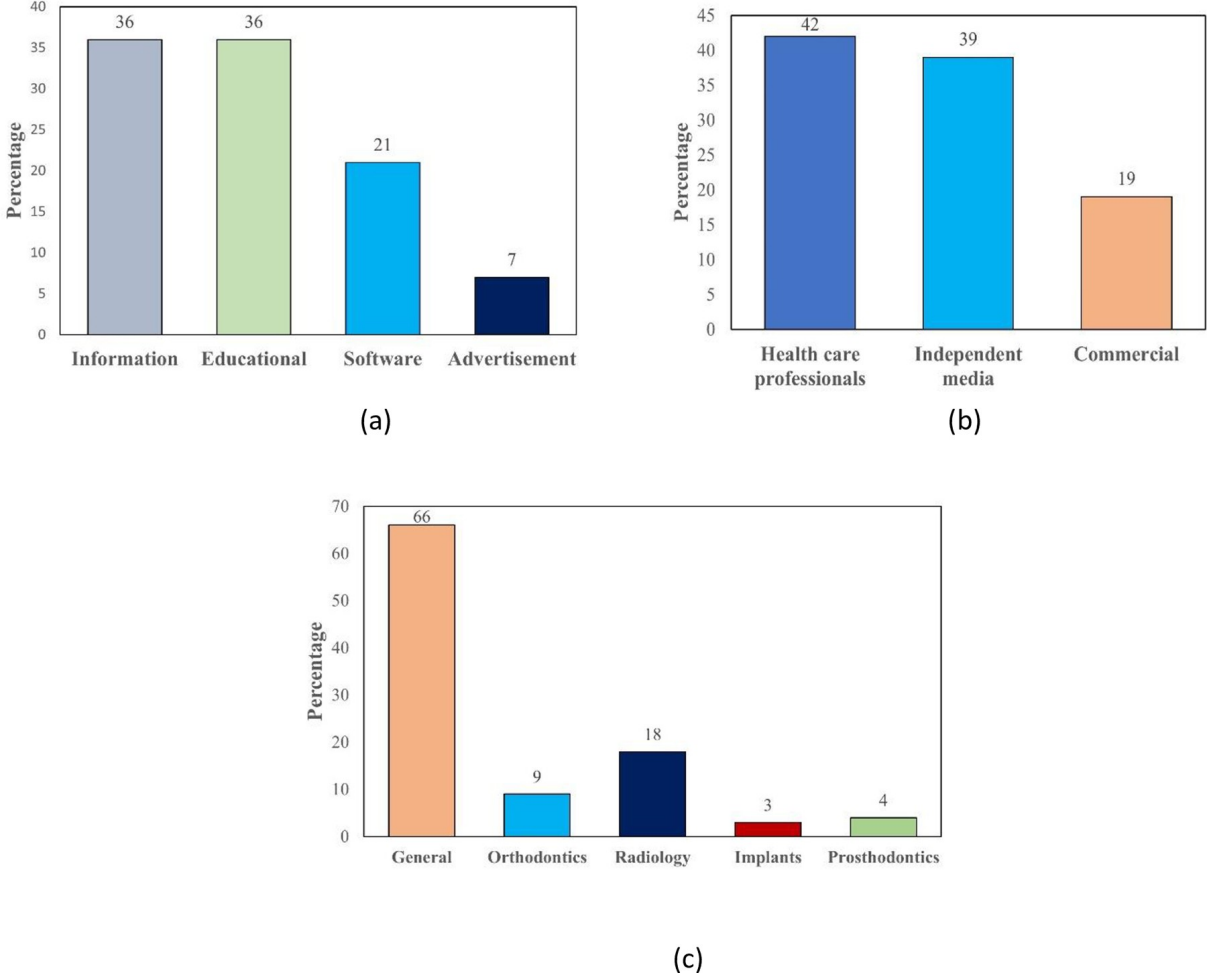
The percentage of videos based on (a) type of content (b) source of the video (c) type of specialty.

According to the analysis of YouTube™ videos, 36% of the content provided information about AI use in dentistry, 21% specifically discussed software, and 36% were educational. Regarding the source of the video, 43% were uploaded by healthcare professionals, 39% from independent media, and 19% were commercial. According to **Fig 2**, videos on general dentistry topics made up 66% of the content, followed by radiology at 18%, orthodontics at 9%, prosthodontics at 4%, and implants at 3%.

The quality and reliability of YouTube™ videos on AI use in dentistry vary by source and content. Videos uploaded by healthcare professionals scored highest on DISCERN (58.88, p<0.05) and mGQS (3.95, p<0.05), indicating higher quality and reliability compared to independent media and commercial sources. Educational videos scored the highest across all three assessment tools: DISCERN (57.85), mGQS (4.03), and JAMA (3.40). Videos focusing on AI-related information scored lower on JAMA (2.61, p<0.05) compared to educational videos, as well as lower on both DISCERN (48.22) and mGQS (3.15). The specialty of the video showed no significant differences in quality and reliability scores, suggesting content type and source are more critical factors (**Table 2**).

**Table 2:** Quality and reliability scores of reviewed YouTube videos according to video source and content.

		DISCERN		JAMA		mGQS	
Source of the video		Mean (SD)	*P* value (F-Value)	Mean (SD)	*P* value (F-Value)	Mean (SD)	*P* value (F-Value)
	Health care professionals	58.88(10.24)	<0.05* (6.5)	2.91(0.84)	<0.05* (4.3)	3.95(0.67)	<0.05* (3.4)
	Independent media	53.01(11.16)		2.54(0.64)		3.67(0.91)	
	Commercial	48.32(10.36)		2.62(0.82)		3.35(0.78)	
Video content	Information on AI	55.15(11.28)	<0.05* (3.5)	2.61(0.65)	0.10* (2.3)	3.83(0.73)	<0.05* (6.5)
	Educational	57.85(9.25)		3.02(0.789)		4.03(0.66)	
	Software	47.82(11.50)		2.29(0.63)		3.11(0.93)	
	Advertisement	56.00(13.97)		2.92(1.15)		3.50(0.63)	
Specialty	General	54.54(10.71)	0.97 (0.05)	2.73(0.81)	0.96 (0.4)	3.81(0.70)	0.74 (0.5)
	Orthodontics	55.25(11)		2.66(0.55)		3.69(0.88)	
	Radiology	55.28(14.04)		2.63(0.83)		3.50(1.14)	
	Implants	52.17(16.92)		2.83(0.28)		3.50(1.32)	
	Prosthesis	54.50(8.81)		2.75(0.86)		3.75(0.64)	

*Significant

**Fig 3:**
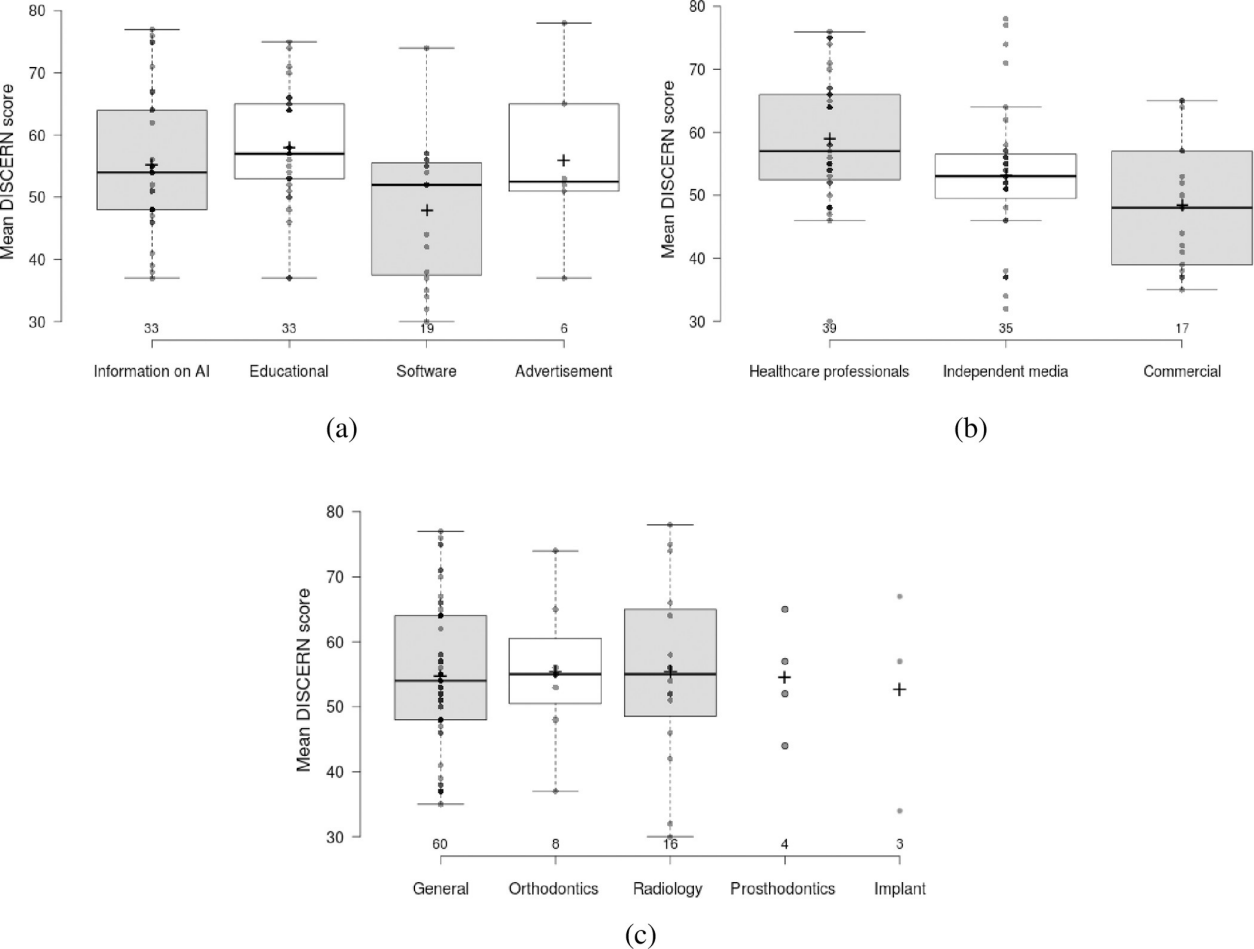
Box plot presentation of mean scores of DISCERN scores according to a) video content, b) video source, c) video on specialty.

The **Fig 3** illustrates variation in the mean of DISCERN scores for YouTube™ videos type category. The ICC value indicates perfect reliability for DISCERN 0.95(0.91–0.97), JAMA 0.895(0.77–0.91) and for mGQS 0.81(0.79–0.84).

The analysis reveals a positive trend in the characteristics of AI-related videos over time. Scores from DISCERN, mGQS, and JAMA steadily increased, indicating improvements in the consistency, comprehensiveness, and presentation of information. Like ratios exhibit noticeable variation, with 2024 showing 4.55(6.67), suggesting exceptionally engaging content compared to prior years (Table 3).

**Table 3:** Mean (SD) of DISCERN, mGQS, and JAMA scores across years for AI-related video content.

Year	Number of videos	DISCERN	mGQS	JAMA	Number of views	Like ratio
2017	1	46	3.50	2.50	1100	2.27
2018	2	51.25(5.30)	3.75(0.35)	2.13(0.18)	2976.50(3144.50)	1.20(0.37)
2019	10	53.35(7.03)	3.55(0.72)	2.60(0.57)	1557.20(2558.72)	1.82(3.11)
2020	9	58.44(12.48)	3.67(0.75)	2.56(0.68)	2801.00(7419.29)	1.50(1.15)
2021	22	56.23(9.59)	3.73(0.81)	2.51(0.50)	2565.27(4204.31)	1.27(1.42)
2022	21	56.31(12.33)	3.83(1.02)	2.65(0.61)	914.48(2188.26)	1.49(1.45)
2023	19	51.16(12.70)	3.68(0.80)	2.95(1.03)	1430.05(1608.75)	2.29(2.40)
2024 (Up to Study Date)	7	53.43(14.28)	3.93(0.79)	3.43(1.27)	781.86(781.58)	4.55(6.67)

The correlation table shows views and likes have a moderate positive correlation (0.266). suggesting that videos with more views tend to receive more likes. Views and subscribers also show a moderate positive correlation (0.217), indicating that videos with higher views may attract more subscribers. Video source and DISCREN have a strong correlation (0.758). Video content and DISCREN have a strong positive correlation (0.606), implying that certain types of video content are associated with higher DISCREN scores. DISCERN and JAMA displayed a moderately significant correlation (0.61). mGQS showed a minimal correlation with both DISCERN (−0.04) and JAMA (0.16). mGQS and JAMA exhibit a strong positive correlation (0.613), indicating that these variables show similar associations with other factors (**Table 4**).

**Table 4:** Correlation matrix between the variables^a^.

Length of video	1	-0.02	0.26*	0.07	-0.02	-0.11	-0.07	0.60**	0.48**	0.58**
Views	-0.02	1	0.12	0.21*	-0.07	-0.11	-0.07	0.01	0.19	0.02
likes	0.26*	0.12	1	0.08	-0.06	-0.15	-0.02	0.53*	0.21*	0.36**
subscribers	0.07	0.21*	0.08	1	0.16	-0.19	0.03	0.11	0.02	0.00
Specialty	-0.02	-0.07	-0.06	0.16	1	0.07	0.41**	0.00	-0.11	-0.01
Source video	-0.11	-0.11	-0.15	-0.19	0.07	1	0.34**	0.75**	0.67**	0.77*
Video content	-0.07	-0.07	-0.02	0.03	0.41**	0.34**	1	0.54	0.66*	0.32
DISCREN	0.60**	0.01	0.53*	0.11	0.00	0.75**	0.54	1	-0.04	0.61**
mGQS	0.48**	0.19	0.21*	0.02	-0.11	0.67**	0.66*	-0.04	1	0.16
JAMA	0.58**	0.02	0.36**	0.00	-0.01	0.77*	0.32	0.61**	0.16	1
	Length of video	Views	likes	subscribers	Specialty	Source video	Video content	DISCREN	mGQS	JAMA

^a^Pearson correlation matrix.

*p < 0.05.

**p < 0.01**

## Discussion

YouTube™ is one of the most well-known social media platforms is a free video-sharing website. It offers a wide range of videos covering topics such as identifying treating and preventing diseases [34]. The present study evaluated the quality of information on YouTube™ regarding the use of AI in dentistry. The study showed YouTube™ has informative and moderately reliable videos on AI in dentistry. This study sheds light on the quality and reliability of YouTube™ video content related to AI in dentistry.

Our study’s results indicate that YouTube™ serves as a moderate source of information on AI in dentistry as assessed by DISCERN, JAMA and mGQS. Several studies have developed content scores using GQS or JAMA to measure the quality of the videos [35–38]. These findings align with previous studies on YouTube™ academic source videos. demonstrating good reliability and quality [34, 36]. Certain studies have identified low-quality videos on YouTube^TM^ and recommended implementing peer review procedures and scientific evaluation processes before content is uploaded [37, 39].

AI applications can be divided into two main categories: virtual and real-world, Machine learning, including deep learning, belongs to the virtual category. It uses mathematical algorithms to improve learning by incorporating experience [40]. The second type of medical AI involves physical items, medical equipment, and advanced robots, often referred to as carebots. which are used to provide treatment [41]. AI in dentistry is used to identify healthy and diseased structures, diagnose disorders and predict treatment outcomes. It is also widely used in dental labs and increasingly integrated into dental education. AI applications in dentistry include caries detection, radiology, orthodontics, and implants [42].

Most of the videos in our study focused on AI in dentistry, explaining the basics of AI and how it can be used in dental practice. Dentists can use AI systems to improve the accuracy of diagnosis, patient management and treatment outcome prediction. YouTube™ videos on AI in dentistry revealed interesting content trends. Around 36% of the content was educational, informing viewers about the use of AI in dentistry. A previous study analyzed YouTube™ videos as a source of information on AI in medicine and found that 40% of the videos provided educational content, while the rest focused on specific AI tools and software [43].

The most common specialty-based videos in our study were related to radiology. A previous study examined the popularity, subject, reliability and educational value of YouTube™ videos on AI in dental radiology. The results revealed that YouTube™ hosts credible and high-quality videos that dentists can utilize to gain insights into AI use in dental radiology [36]. The videos explained AI models based on X-ray and cone beam computed tomography (CBCT) images used to address various clinical problems. The development of 3D image-based AI systems for automatic detection and classification, treatment planning and prediction includes image files from CBCT and other sources.

The most common specialty-based videos focused on AI use in orthodontics. While no previous studies have specifically examined AI in orthodontics, some studies analyzed YouTube™ videos on orthognathic surgery and highlighted the substandard quality of certain videos [42]. The study indicated that the videos showing AI automatically identifying cephalometric landmarks was moderate in quality. Cephalometric analysis is typically conducted manually or digitally in clinical practice using specialized software. These AI models are used to plan orthodontic treatment more effectively.

A previous study assessed the accuracy of dental implant-related information accessible on YouTube™. Seventy-four videos were analyzed. with usefulness ratings ranging from 0 to 2 and an average rating of 0.324. The study underscored patients’ restricted access to YouTube™ videos about dental implants, noting that many of these videos overlooked crucial dental implant-related issues [39].

Our findings suggest a need to raise awareness among the public, patients and physicians that not all YouTube^TM^ videos on AI in dentistry meet scientific standards. A prior study revealed that healthcare agencies and organizations have published only a few educational and scientifically acceptable medical videos [9]. Only 27% of medical videos were informative and educational tend to be less appealing to laypeople [29].

The correlation between video source and DISCREN was high in this study. Videos produced by healthcare professionals received higher scores for quality, accuracy and informativeness. The quality of independent media varies, leading to more fluctuation in DISCERN scores. Depending on their production value, commercial uploads often feature high-quality visuals, which can boost their scores. Previous studies have explored that healthcare professionals’ social media use influences the quality and reliability of online information [44].

AI’s applications in dentistry extend beyond diagnosis and treatment planning to include personalized patient care, predictive analysis and the automation of routine tasks such as appointment scheduling and follow-up. These broader uses of AI are gradually being integrated into dental practice, promising to revolutionize the field. However, the educational content on YouTube™ concerning these advancements remains limited, with most videos focusing on AI’s basic principles. This gap underscores the importance of producing peer-reviewed, high-quality educational videos that reflect the latest technological innovations and clinical practice.

Dental educators could create evidence-based, informative content, practitioners offer practical insights from experience, and patients provide feedback on clarity and relevance. Collaboration among these stakeholders ensures that AI-related dental content on YouTube is accurate, high-quality, engaging, and accessible to diverse audiences.

A significant limitation of this study is that the outcomes may vary depending on the search terms used, as well as the date and time of the search. The selection of the top 60 results may favor content driven by viewer engagement metrics rather than objective educational quality. The assessment was conducted on a single type of website during a specific period, yet website information constantly evolves. Our study may have overlooked the public’s perspective, as the assessment was conducted by professionals. Only English-language videos were selected, which could limit the scope of the findings. Lack of detailed information about the specific AI tools mentioned in some videos may have impacted the scoring of some videos. This study did not account for video production quality, including factors like visuals, editing style and presentation quality. Which may influence viewers’ perceptions of reliability independently of the content’s factual accuracy or quality. The study assumes access to YouTube from a single country domain. However, recommendation algorithms may influence video promotion based on geographical location. Future research should consider how regional differences in recommendations could affect video selection and overall findings. The authors’ specialties may have influenced video selection and evaluation, potentially introducing bias into the study.

In the future, it is recommended that dental professionals contribute more educational videos on YouTube™. Maintaining up-to-date and reliable information on YouTube™ is crucial, given the rapid development of AI in dental technology. More research is also advised to determine how different audiences and professionals perceive videos on AI use in dentistry. Dental professionals are encouraged to use high-quality visuals, maintain clear and engaging explanations, and collaborate with experts to enhance credibility. Professionals should adhere to ethical standards, avoid promotional content, and focus on patient-centered educational material that reflects the latest advancements in AI and dentistry.

## Conclusion

YouTube™ videos on artificial intelligence in dentistry, as assessed by dental professionals, provide moderately reliable data. Dental students, dentists and patients can use these videos to learn and educate others about AI in dentistry. Although not all content on YouTube™ related to AI in dentistry is reliable, the platform still holds the potential for disseminating knowledge. Healthcare professionals should be mindful of online videos’ impact on patients’ perceptions. It is recommended that professionals upload more videos on AI in dentistry to help ensure the reliability and authenticity of the video content.

## Supporting information

S1 Data Data used for analysis. (XLSX)

S2 Data Tools used for analysis. (DOCX)
