# Supplementary material for: Artificial intelligence in dentistry: Assessing the informational quality of YouTube videos
Source: PLoS One. 2025 Jan 2;20(1):e0316635. doi: 10.1371/journal.pone.0316635 (PMC11695022; doi:10.1371/journal.pone.0316635)
Supplement: S2 Data — (DOCX) [file pone.0316635.s002.docx]

**DISCERN Tool**

|  | Strongly No | No | Partially | Yes | Strongly yes |
| --- | --- | --- | --- | --- | --- |
| **Assessment criteria** |  |  |  |  |  |
| 1. Is the publication reliable? |  |  |  |  |  |
| 2. Does it achieve its aims? |  |  |  |  |  |
| 3. Is it relevant? |  |  |  |  |  |
| 4. Is it clear what sources of information were used to compile the publication (other than the author or producer)? |  |  |  |  |  |
| 5.Is it clear when the information used or reported in the publication was produced? |  |  |  |  |  |
| 6. Is it balanced and unbiased? |  |  |  |  |  |
| 7. Does it provide details of additional sources of support and information? |  |  |  |  |  |
| 8.Does it refer to areas of uncertainty? |  |  |  |  |  |
| 9.Does it describe how each treatment works? |  |  |  |  |  |
| 10.Does it describe the benefits of each treatment? |  |  |  |  |  |
| 11.Does it describe the risks of each treatment? |  |  |  |  |  |
| 12.Does it describe what would happen if no treatment is used |  |  |  |  |  |
| 13.Does it describe how the treatment choices affect overall quality of life? |  |  |  |  |  |
| 14. Is it clear that there may be more than one possible treatment choice? |  |  |  |  |  |
| 15.Does it provide support for shared decision-making? |  |  |  |  |  |
| 16. Based on the answers to all of the above questions, rate the overall quality of the publication as a source of information about treatment choices |  |  |  |  |  |

**JAMA Tool**

| Authorship | Authors and contributors, their affiliations, and relevant credentials should be provided | 1 |
| --- | --- | --- |
| Attribution | References and sources for all content should be listed clearly, and all relevant copyright information noted. | 2 |
| Disclosure | Web site "ownership" should be prominently and fully disclosed, as should any sponsorship, advertising, underwriting, commercial funding arrangements or support, or potential conflicts of interest. This includes arrangements in which links to other sites are posted as a result of financial considerations. Similar standards should hold in discussion forums. | 3 |
| Currency | Dates that content was posted and updated  should be indicated. | 4 |

**Modified Global Quality Score(mGQS)**

| Poor quality, poor flow of the site, most information missing, not at all useful for patients | 1 |
| --- | --- |
| Generally poor quality and poor flow, some information listed but many important topics missing, of very limited use to patients | 2 |
| Moderate quality, suboptimal flow, some important information is adequately discussed but others poorly  discussed, somewhat useful for patients | 3 |
| Good quality and generally good flow, most of the relevant information is listed,  but some topics not covered, useful for patients | 4 |
| Excellent quality and excellent flow, very useful for patients | 5 |
